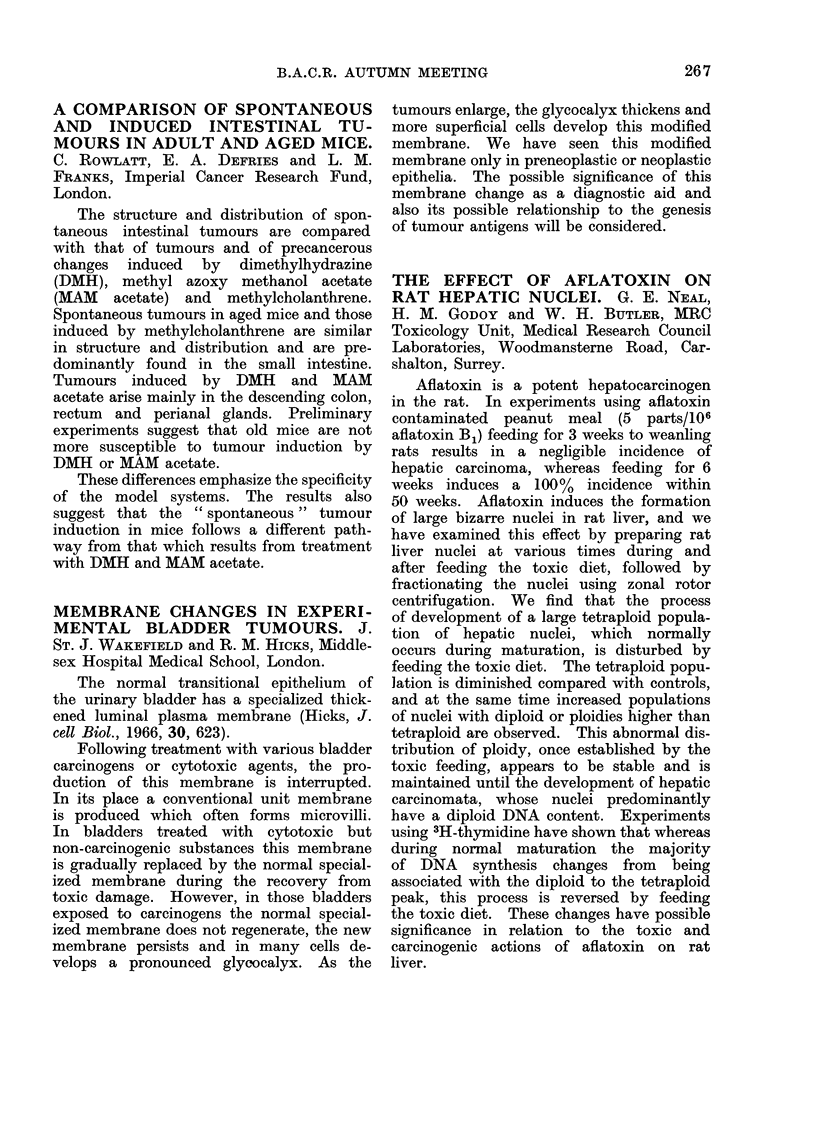# Proceedings: Membrane changes in experimental bladder tumours.

**DOI:** 10.1038/bjc.1975.60

**Published:** 1975-02

**Authors:** S. J. Wakefield, R. M. Hicks


**MEMBRANE CHANGES IN EXPERIMENTAL BLADDER TUMOURS.** J. ST. J. WAKEFIELD and R. M. HICKS, Middlesex Hospital Medical School, London.

The normal transitional epithelium of the urinary bladder has a specialized thickened luminal plasma membrane (Hicks, *J. cell Biol.*, 1966, **30**, 623).

Following treatment with various bladder carcinogens or cytotoxic agents, the production of this membrane is interrupted. In its place a conventional unit membrane is produced which often forms microvilli. In bladders treated with cytotoxic but non-carcinogenic substances this membrane is gradually replaced by the normal specialized membrane during the recovery from toxic damage. However, in those bladders exposed to carcinogens the normal specialized membrane does not regenerate, the new membrane persists and in many cells develops a pronounced glycocalyx. As the tumours enlarge, the glycocalyx thickens and more superficial cells develop this modified membrane. We have seen this modified membrane only in preneoplastic or neoplastic epithelia. The possible significance of this membrane change as a diagnostic aid and also its possible relationship to the genesis of tumour antigens will be considered.